# Methylene Blue injection via superior mesenteric artery microcatheter for focused enterectomy in the treatment of a bleeding small intestinal arteriovenous malformation

**DOI:** 10.1186/1749-7922-9-17

**Published:** 2014-02-19

**Authors:** James Frydman, Hany Bahouth, Maxim Leiderman, Amos Ofer, Yoram Kluger

**Affiliations:** 1 Department of General Surgery, Rambam Health Care Campus, P.O. B 9602, 31096 Haifa, Israel; 2 Depatment of Radiology, Rambam Health Care Campus, P.O. B 9602, 31096 Haifa, Israel

**Keywords:** Obscure gastrointestinal bleeding, Computed tomography angiography, Super-selective angiography, Intra-operative methylene blue injection

## Abstract

**Introduction:**

Obscure gastrointestinal bleeding from the small intestine may present the Acute Care Surgeon with a formidable diagnostic and therapeutic challenge. Despite the current array of diagnostic studies, localization of the causative pathology may be elusive, especially when the bleeding is intermittent. When a small intestinal arteriovenous malformation is the responsible lesion, a technique combining super-selective angiography with intra-operative methylene blue injection and focused enterectomy has been described in a number of case series. The current case report utilizes this same approach with emphasis on computed tomography angiography representing a key first step in the diagnostic algorithm.

**Case report:**

In this case report, we describe the diagnosis and treatment of obscure gastrointestinal bleeding emanating from an arteriovenous malformation in the small intestine of a 52 year old male. After an extensive work-up including upper and lower endoscopy, double balloon enteroscopy and capsule endoscopy, he was referred for computed tomography angiography. Though he was not actively bleeding, a jejunal arteriovenous malformation was localized on imaging. This prompted directed transfemoral angiography, placement of a super-selective microcatheter in the 4th jejunal arterial branch, intra-operative methylene blue injection and focused enterectomy with pathological confirmation. The patient was found to be free of gastrointestinal bleeding on 6 month follow-up.

**Conclusions:**

A step-wise, rational diagnostic approach should be utilized in the evaluation of obscure gastrointestinal bleeding. In the non-actively bleeding patient, computed tomography angiogram may facilitate the diagnosis of a small intestinal arteriovenous malformation. Methylene blue injection via a super-selective angiographic microcatheter may then allow for focused enterectomy.

## Introduction

Gastrointestinal (GI )bleeding from the small intestine remains a formidable diagnostic and therapeutic challenge for the Acute Care Surgeon. This is secondary to its length and mobility, as well as relative inaccessibility [1]. While the stomach, duodenum, and colon are comparatively fixed in location and evaluable by means of conventional upper and lower endoscopy, the diagnosis of bleeding from the jejunum and ileum may require a series of alternative tests. This may include, but not limited to, small bowel enteroscopy, double balloon enteroscopy, capsule endoscopy, nuclear medicine bleeding scan, computed tomography angiography (CTA), invasive angiography, or exploratory laparotomy with intraoperative enteroscopy.

In a landmark paper in 1978, Fogler and Golembe described the injection of methylene blue through direct cannulation of the superior mesenteric artery in the operating theater, guided by preoperative angiographic findings of an arteriovenous malformation (AVM) in the proximal jejunum. A segment of small bowel measuring 10 cm which *cleared* the blue dye rapidly while the color remained in proximal and distal segments was presumed to contain the pathological AVM. Though this was not demonstrated on pathology, the patient remained free of GI bleeding on 6 month follow-up [2].

From this highly invasive and non-selective approach, several refinements on this technique have been pioneered over the years to result in a less invasive and more focused surgical resection in the treatment of GI bleeding from the small intestine [3-8]. In this report, we describe how pathological findings on CTA in a non-actively, obscure GIB patient prompted super-selective angiographic catheter placement and, ultimately, limited enterectomy directed by intra-operative methylene blue injection.

## Case report

The patient is a 52 year-old male with past medical history significant for coronary artery disease, hyperlipidemia, gout and obesity. He had undergone cardiac catheterization and stent placement 4 years ago and continued on anti-platelet therapy with aspirin and Plavix. Two years prior to current presentation, he underwent work-up for melanotic stools with upper, lower and capsule endoscopy. He was diagnosed at that time with duodenitis, attributed to Arcoxia, a COX-2 inhibitor he had been prescribed for treatment of gouty arthritis, with likely synergistic effect due to concomitant aspirin intake. Past surgical history was notable for laparoscopic sleeve gastrectomy earlier this year with resultant 35 kilogram weight loss.

His current presentation was marked by intermittent melanotic stools, fall in hemoglobin to a low of 7.3 g/dl and orthostatic symptoms. He was resuscitated and required a blood transfusion. Nasogastric tube placement did not reveal evidence of bleeding. Further work-up included upper and lower endoscopy which failed to reveal the source of bleeding. Capsule endoscopy, however, showed active bleeding localized to the jejunum, which prompted small bowel enteroscopy, which failed to show pathology to a depth of 160 cm. This was followed by double balloon enterosopy to a depth of 2m reaching the ileum. Again, this was negative for any responsible lesions. At this time, we elected to perform CTA of the abdomen both to exclude a mass lesion and attempt to localize a possible AVM. Of note, the patient was not experiencing any active bleeding at this time.

**Figure 1:**
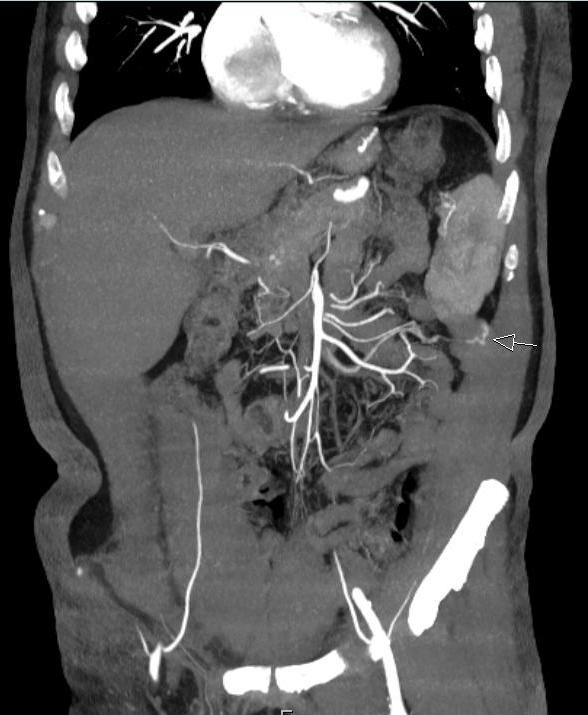
**CTA - Coronal reconstruction with a slab of 1 cm.** Abnormal vessel (AVM) (arrow) from a small jejunal branch of SMA.

**Figure 2:**
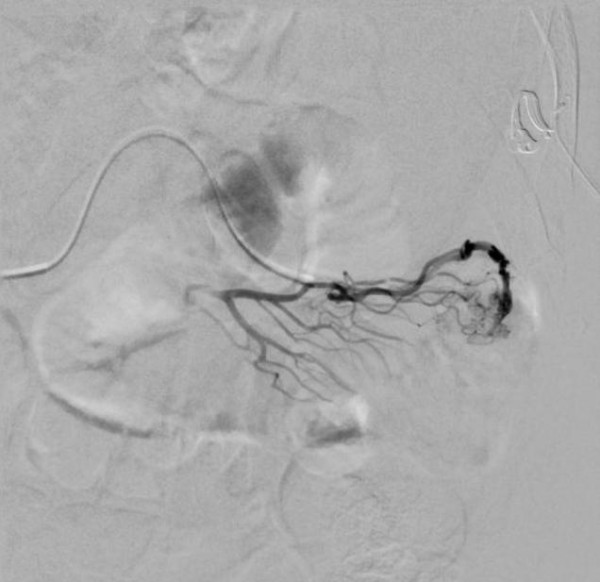
Transfemoral angiography - selective injection of 4th jejunal branch through a 2.7 Fr microcatheter.

In the selected cut from his CTA (Figure 1), an ectatic, antimesenteric blood vessel was noted adjacent to a loop of jejunum in the left side of the abdomen. This appeared to be an anomalous AVM as evidenced by early filling of an associated vein on arterial phase. Also notable was the finding of replaced left *and* right hepatic arteries. Given the CTA findings, he was referred for angioembolization. During this procedure, the visualized fourth jejunal branch from the superior mesenteric artery appeared to give rise to the AVM seen on CTA (Figure 2). This was cannulated distally with a super-selective 2.7 Fr microcatheter, but the lesion was not amenable to embolization given robust collateralization. The decision was made to leave the micro-angiocatheter in-situ to facilitate intraoperative identification of the small intestinal AVM. The sheath and catheter were secured at the groin entry site, 2500 units of heparin were administered intravenously and the patient was transported directly to the operating theater.

**Figure 3:**
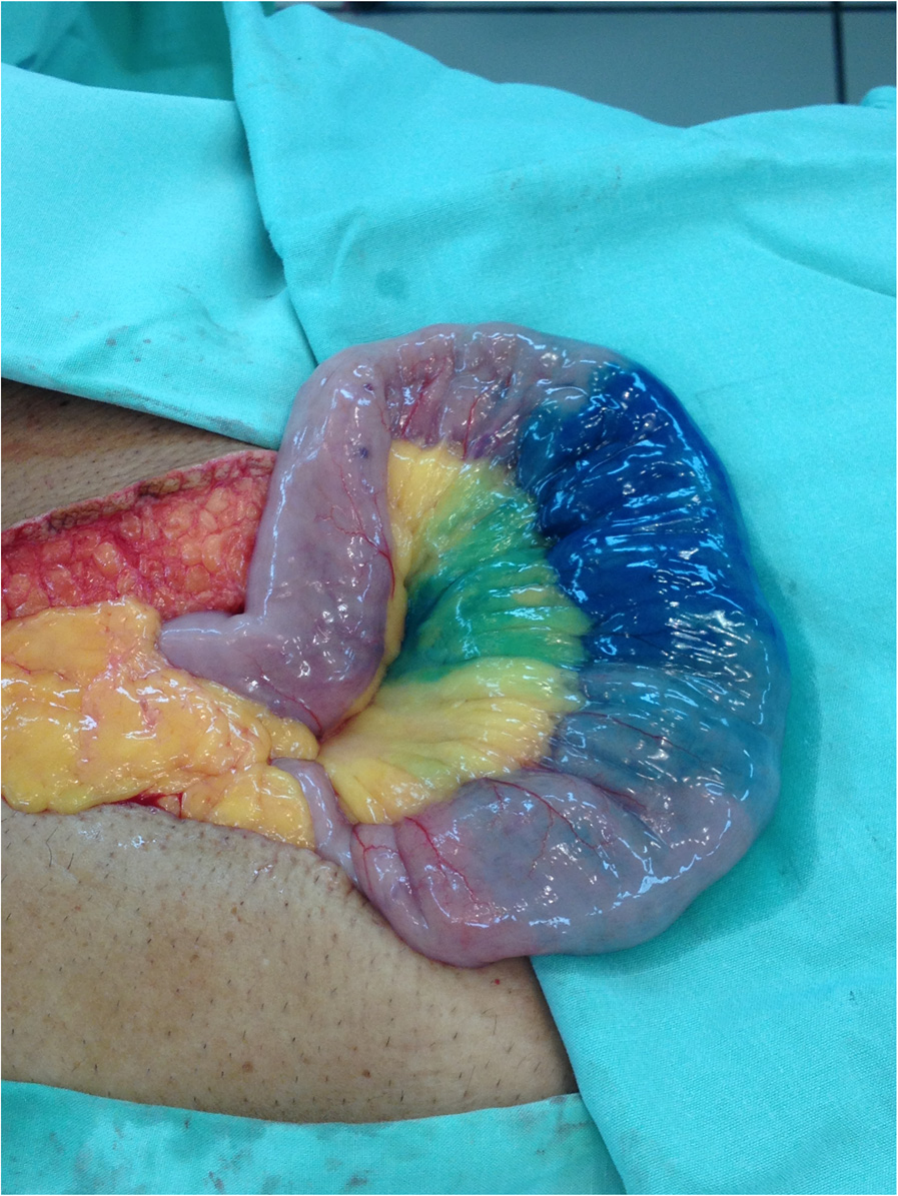
Intraoperative demonstration of methylene blue staining of affected small bowel segment containing the AVM.

A limited midline incision was utilized to gain access into the peritoneal cavity and expose the small intestine. Two mL of dilute methylene blue were then injected via the super-selective angiographic microcatheter, immediately staining a 10 cm segment of the distal jejunum and corresponding mesentery (Figure 3). A segmental small bowel resection was performed. The patient had an unremarkable post-operative course and pathology demonstrated angiodysplasia in the small bowel segment with clean margins. At 6 month telephone follow-up the patient is doing well and denies any further episodes of melena.

## Discussion

Obscure GI bleeding has been defined as bleeding which persists or recurs after upper and lower endoscopy and radiographic evaluation of the small bowel. Comprising up to 5% of cases of GI bleeding with 75% of them localizing to the small intestine [9], these patients may require multiple blood transfusions and be subject to a battery of repeat diagnostic studies before definitive diagnosis is accomplished. The most likely etiologies are broken down by age group. In patients younger than 40, the most likely lesions include Meckel’s diverticulum, inflammatory bowel disease, or a small bowel tumor such as a gastrointestinal stromal tumor (GIST), lymphoma, carcinoid, polyp or adenocarcinoma. In contrary, patients older than 40 are most likely to have bleeding from vascular anomalies, erosions or NSAID-related ulcerations. Overall, vascular lesions comprise 40% of all causes [10].

Two years after Fogler and Golembe’s description of open cannulation of the superior mesenteric artery, Athanasoulis and colleagues in 1980 described the placement of super-selective angiographic catheters and intraoperative methylene blue injection to localize and treat two patients with AVMs and one with ulcerations in the small intestine [3]. Refinements on the technique have been described in subsequent reports which have paralleled advancement in angiographic methods, including provocative angiography with fibrinolytic agents [4-8].

From these reports, several guiding principles can be elucidated. When the AVM is localized on angiography, the most distal arterial tributary should be cannulated by a microcatheter and safely secured for transport. This can be done in the angiography suite or a hybrid operating theater. Following this the small bowel must be exposed either via a limited midline laparotomy or laparoscopy *before* injection of methylene blue. The limited segment of small bowel, usually 10cm or less is readily identified and resected with pathological confirmation. Clinical success is confirmed by long-term follow up.

After a careful review of the literature, this report represents the first case in the utilization of CTA in the diagnosis of a non-actively bleeding small bowel AVM which then enabled focused angiography and subsequent limited enterectomy. The CTA demonstrated the abnormality in the left-sided, proximal jejunum which corresponded to the 4th jejunal branch by transfemoral angiography. Not only did this spare the patient additional contrast load, it may have not been localized, or required provocative angiography, with its inherent risks, if not for the pathological finding on CTA. As the quality of the CTA has improved with new generation scanner technology, this diagnostic study should be considered in the work-up of the non-actively, obscure GI bleeding patients, with a focus on small bowel lesions and AVMs. Further study is warranted to truly gauge its sensitivity and specificity in this patient population.

## Consent

Written informed consent was obtained from the patient for publication of this Case Report and any accompanying images. A copy of the written consent is available for review by the Editor-in-Chief of this journal.

## Competing interests

The authors do not have any financial or non-financial competing interests to declare.

## Authors’ contributions

Study concept and design: JF, HB, YK. Acquisition of data: JF, HB, ML, AO. Analysis of data: JF, HB, ML, AO, YK. Drafting of manuscript: JF. Critical revision of manuscript: YK. Study supervision: AO, YK. All authors read and approved the final manuscript.
